# Draft Genome Sequences of Leptospira interrogans Serovar Copenhageni Strains Isolated from Patients with Weil’s Disease in Brazil

**DOI:** 10.1128/MRA.01538-19

**Published:** 2020-02-13

**Authors:** Camilla N. R. Trindade, Pedro H. N. Panzenhagen, Ricardo M. Junqueira, Deyse C. V. Silva, Carlos A. Conte-Junior, Ilana T. Balassiano

**Affiliations:** a Bacterial Zoonosis Laboratory, Oswaldo Cruz Institute, Oswaldo Cruz Foundation, Rio de Janeiro, Brazil; b Chemistry Institute, Department of Chemistry, Federal University of Rio de Janeiro, Rio de Janeiro, Brazil; University of Arizona

## Abstract

Leptospirosis is a worldwide zoonosis caused by pathogenic species of *Leptospira*. In Brazil, this disease is endemic, presenting epidemic potential in rainy seasons. Here, we announce the whole-genome sequences of two L. interrogans serovar Copenhageni strains isolated from blood samples from two icteric patients associated with severe leptospirosis in Brazil.

## ANNOUNCEMENT

Leptospirosis is a worldwide zoonosis caused by pathogenic serovars of *Leptospira* species. The genus *Leptospira* can be categorized into three subgroups, saprophyte (L. meyeri, L. yanagawae, L. terpstrae, L. biflexa, L. vanthielli, and L. wolbachii), intermediate (L. licerasiae, L. wolffii, L. fainei, L. broomii, and L. inadai), and pathogenic (L. interrogans, L. kirschneri, L. noguchii, L. borgpetersenii, L. mayottensis, L. santarosai, L. weilii, L. alexanderi, L. alstonii, and L. kmetyi), and subdivided into more than 200 serovars (1, 2). Leptospirosis produces a wide variety of clinical manifestations, ranging from subclinical infection to severe and fatal disease, and causes annually 1 million cases in humans and an estimated 58,900 deaths (3). This disease is more common in tropical regions and is found predominantly in impoverished populations in developing countries, where incidence peaks are observed during the rainy season. In Brazil, this infection is endemic and is recognized as a disease with epidemic potential and a significant impact on public health. In humans, severe leptospirosis cases in urban areas are frequently associated with L. interrogans serovar Copenhageni (4). In this context, we sequenced two *Leptospira* strains belonging to the Leptospira Collection (Coleção de Leptospira [CLEP]/Oswaldo Cruz Institute-Fiocruz) which were isolated from blood samples from two icteric patients associated with severe leptospirosis in Brazil. The study protocol was reviewed and approved by the Scientific Review Board of the Oswaldo Cruz Institute-Fiocruz, Rio de Janeiro, Brazil (Presentation Certificate for Ethical Appreciation 86308318.8.0000.5248).

Strains CLEP00152 and CLEP00179 (L. interrogans serovar Copenhageni) were cultured in Ellinghausen-McCullough-Johnson-Harris (EMJH) liquid medium supplemented with bovine serum albumin at 28^º^C for 7 to 10 days under aerobic conditions, and the DNA was obtained with the DNeasy blood and tissue kit (Qiagen). Libraries were prepared with the Nextera XT DNA library preparation kit (Illumina) and sequenced on a HiSeq 2500 system (Illumina) to obtain 2 × 100-bp reads. The read quality was accessed utilizing FastQC v0.11.5 (5). The reads were processed with Trimmomatic v0.36 (6), and overlapping reads were extended using FLASH v1.2.11 (7). The processed reads were assembled using SPAdes v3.11.1 (8). The genome statistics were accessed with QUAST v4.6.0 (9), and finally, the contigs were annotated with NCBI Prokaryotic Genome Annotation Pipeline (PGAP) (10). Default parameters were used for all software.

The assembly of strain CLEP00152 generated 176 contigs, which covered a total of 4,583,061 bp with 42,808,073 paired-end reads, an *N*_50_ value of 83.218 bp, and an average coverage of 888×. The assembly of strain CLEP00179 generated 158 contigs, which covered a total of 4,581,547 bp with 7,072,704 paired-end reads, an *N*_50_ value of 89.408 bp, and an average coverage of 152×. The G+C content average was 34.99% for both strains. The genomes sequenced are each composed of approximately 3,600 coding sequences (CDSs), 40 tRNAs, and 1 CRISPR. This genomic study will provide a better understanding of the Brazilian serovar Copenhageni strains and their relationship with the diverse group of pathogenic *Leptospira* spp.

### Data availability.

The whole-genome sequencing projects for Leptospira interrogans strains CLEP00152 and CLEP00179 have been deposited in NCBI GenBank under the accession numbers VWOW00000000 and WNNH00000000, respectively. The raw Illumina HiSeq data have been deposited in the NCBI Sequence Read Archive (SRA) under the BioProject accession number PRJNA560059.
